# Gdown1 Associates Efficiently with RNA Polymerase II after Promoter Clearance and Displaces TFIIF during Transcript Elongation

**DOI:** 10.1371/journal.pone.0163649

**Published:** 2016-10-07

**Authors:** Elizabeth DeLaney, Donal S. Luse

**Affiliations:** Department of Cellular and Molecular Medicine, Lerner Research Institute, Cleveland Clinic, Cleveland, Ohio, United States of America; Hong Kong University of Science and Technology, HONG KONG

## Abstract

Pausing during the earliest stage of transcript elongation by RNA polymerase II (Pol II) is a nearly universal control point in metazoan gene expression. The substoichiometric Pol II subunit Gdown1 facilitates promoter proximal pausing in vitro in extract-based transcription reactions, out-competes the initiation/elongation factor TFIIF for binding to free Pol II and co-localizes with paused Pol II in vivo. However, we have shown that Gdown1 cannot functionally associate with the Pol II preinitiation complex (PIC), which contains TFIIF. In the present study, we determined at what point after initiation Gdown1 can associate with Pol II and how rapidly this competition with TFIIF occurs. We show that, as with the PIC, Gdown1 cannot functionally load into open complexes or complexes engaged in abortive synthesis of very short RNAs. Gdown1 can load into early elongation complexes (EECs) with 5–9 nt RNAs, but efficient association with EECs does not take place until the point at which the upstream segment of the long initial transcription bubble reanneals. Tests of EECs assembled on a series of promoter variants confirm that this bubble collapse transition, and not transcript length, modulates Gdown1 functional affinity. Gdown1 displaces TFIIF effectively from all complexes downstream of the collapse transition, but this displacement is surprisingly slow: complete loss of TFIIF stimulation of elongation requires 5 min of incubation with Gdown1. The relatively slow functional loading of Gdown1 in the presence of TFIIF suggests that Gdown1 works in promoter-proximal pausing by locking in the paused state after elongation is already antagonized by other factors, including DSIF, NELF and possibly the first downstream nucleosome.

## Introduction

The promoter proximal pause, in which Pol II typically accumulates at about 50 bp downstream of transcription start, is a universal feature of transcription by RNA polymerase II in metazoans. Promoter proximal pausing plays a critical regulatory role in transcription and disruption of pausing can have widespread deleterious effects on gene expression [1–3]. In a recently reported example, an inhibitor of the Cdk7 subunit of TFIIH was shown to disrupt pausing in part by impairing the recruitment of pause factors to early elongation complexes (EECs) [4]. Treatment of a leukemia cell line with THZ1 resulted in a reduction in Pol II occupancy at promoters and significantly reduced global steady-state mRNA levels [5].

While the DSIF and NELF factors are necessary for pausing, the complete set of pause-inducing factors and their mechanisms of action have yet to be elucidated [1]. The best approximation of the +50 pause in vitro has been observed using transcription with nuclear extracts. Those studies have identified Gdown1 as an important additional component in establishing the promoter proximal pause by Pol II [6]. Gdown1 was originally reported as the 13^th^ subunit of Pol II, present in 30–50% of polymerases isolated from tissue sources [7]. Gdown1 binds tightly to Pol II, even in the presence of high concentrations of salt [6,8] or low levels of urea [7]. In a promoter-independent transcription assay with pure Pol II alone, Gdown1 did not affect elongation rates [7]. However, when Gdown1 was added to extract-based transcription reactions in which escape into productive elongation had been blocked, Gdown1 strongly increased pausing, resulting in promoter proximal accumulation of Pol II [6]. ChIP assays demonstrated that Gdown1 co-localizes with Pol II in the promoter proximal region in vivo; the ratio of Gdown1 to Pol II was found to be highest at poorly expressed genes [6].

Gdown1’s role in pausing is further reinforced by its ability to inhibit the interaction of the general transcription factor (GTF) TFIIF with Pol II [6,8–10]. In addition to its role in PIC assembly (reviewed in [11]), metazoan TFIIF is unique among the GTFs because it also strongly stimulates elongation rates of EECs [12–15]. Initial structural studies suggested that Gdown1 and TFIIF can occupy at least a subset of each other’s binding sites on Pol II [9], which agrees with the ability of Gdown1 to successfully displace TFIIF from both free Pol II and elongation-committed Pol II complexes upon extended incubation [6,8–10]. Since TFIIF is essential for assembly of the Pol II preinitiation complex (PIC), it seemed possible that addition of Gdown1 to PICs might block transcription. However, we were surprised to discover that Gdown1 cannot functionally associate with a preassembled PIC [10].

This last finding raised a series of questions. In order to affect pausing early in transcript elongation, Gdown1 should enter the EEC soon after initiation. The Pol II complex undergoes many transitions as it transforms from a PIC to a fully committed elongation complex. These include template melting to form open complex, extension of the RNA-DNA hybrid to the length typical of EECs (~+8), closure of the upstream segment of the initial transcription bubble (bubble collapse), which marks the beginning of promoter clearance (~+10), and final commitment to a stable elongation complex which no longer backtracks (+25 to +32) (reviewed in [16]). Our previous work suggested that Gdown1 could load rapidly into EECs in the absence of TFIIF, but when Gdown1 is forced to compete with TFIIF already resident in the PIC, displacement might be significantly slower.

In our present study we demonstrate that while Gdown1 ultimately displaces TFIIF from EECs, functional loading of Gdown1 is surprisingly slow. Gdown1 only blocks stimulation of elongation by TFIIF in half of EECs after one minute of incubation with Gdown1. We also found that Gdown1 loading provides a sensitive probe for the transitions that occur in the earliest stages of transcription. Consistent with our previous work, Gdown1 is unable to functionally enter a PIC, and contrary to our initial expectations it is also unable to enter either an open complex or a complex abortively initiating after forming the first bond. While Gdown1 can function with the earliest stable elongation complexes (+5 to +8), it is most effective in blocking elongation stimulation by TFIIF only after Pol II has achieved promoter clearance following the bubble collapse transition. Downstream of that point, complexes are equivalent in their ability to respond to Gdown1. All of our results suggest that Gdown1’s role in early elongation involves the reinforcement of pausing imposed by other factors, rather than the initiation of pausing. This is consistent with the fact that Gdown1 blocks the action of the TTF2 termination factor [6], which would increase the lifetime of promoter proximal paused complexes.

## Results

Earlier studies indicated that Gdown1 can completely displace TFIIF from both free Pol II and Pol II in elongation-committed complexes [6,8–10]. However, Gdown1 cannot enter a PIC, which requires TFIIF for assembly [10]. For Gdown1 to affect early elongation, it should be able to enter EECs and displace TFIIF at or shortly after initiation. We knew from our earlier work that during transcription in vitro under the conditions used here, Pol II alone elongates RNA chains relatively slowly (~90 nt/min), while in the presence of TFIIF, elongation rates are much faster (~400 nt/min) [10,17]. The templates for this study contain short G-less cassettes from 5 to 30 nt in length, allowing us to generate EECs temporarily halted at various points just downstream of transcription start. By challenging these EECs with Gdown1 followed by chase with all four NTPs, we can assess the extent to which Gdown1 has converted the complexes from rapidly elongating (containing TFIIF) to slowly elongating (lacking TFIIF). This approach exploits the fact that TFIIF cannot displace Gdown1 once Gdown1 has bound to Pol II [9].

**Fig 1:**
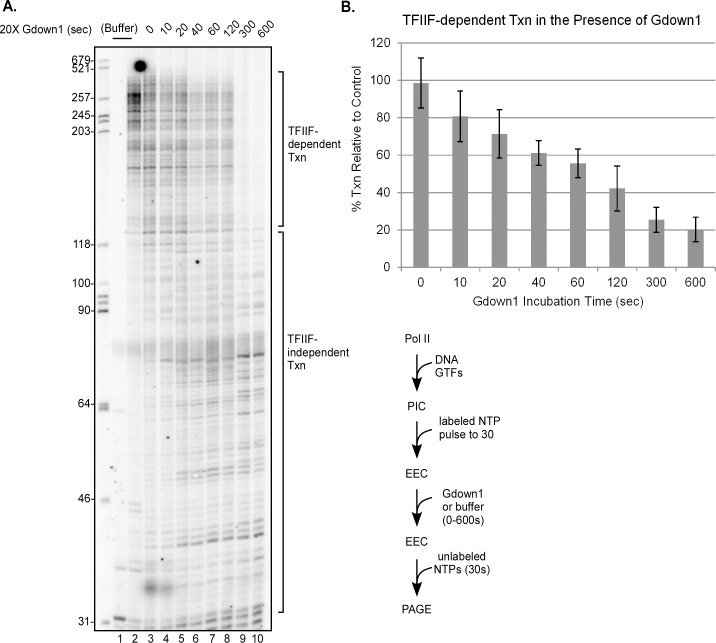
Full functional loading of Gdown1 to EECs takes nearly five minutes. (A) PICs were assembled with TFIIF containing full-length RAP74 in solution on the AdML 31g(M) template [19] and EECs stalled at +30 were generated with ATP, UTP, and [α-^32^P]CTP as described in Materials and Methods. EECs were then incubated with 240 fmol of Gdown1 for 0 to 10 min (except lanes 1 and 2) and chased (except lane 1) for 30 sec with 200 μM NTPs. 240 fmol of Gdown1 is a 20-fold excess over Pol II and a 2-fold excess over TFIIF. DNA size markers were run in the far left lane of the gel and their lengths are indicated. Lengths of RNAs in the chase reactions were quantified as TFIIF-dependent and TFIIF-independent as noted on the right. A schematic of the assay is shown to the lower right of the gel. (B) Average percents of TFIIF-dependent transcription were quantified from results like those shown in panel (A) as described in Materials and Methods. The error bars indicate the mean ± S.D. based on five replicates.

To perform these tests, we assembled PICs with purified (TFIIH) or recombinant (TBP, TFIIB, TFIIF, and TFIIE) human GTFs and purified human Pol II on DNA templates based on the Adenovirus major late (AdML) TATA box promoter. Our Pol II preparation lacks any Gdown1. We began (Fig 1) with EECs halted at +30, since these complexes should be fully committed to elongation [18]. Transcription was initiated with a brief pulse of ATP, limiting levels of UTP and ^32^P-labeled CTP. These EECs were then incubated with a 20-fold excess of Gdown1 for up to ten minutes followed by chase for 30 seconds with unlabeled excess NTPs. As shown in Fig 1A, lane 1, the primary nascent transcript in the EECs was 30 nt, corresponding to the first G stop in the template. The elongation rate seen in the absence of Gdown1 (lane 2) is that expected for TFIIF-stimulated elongation. When Gdown1 was incubated with EECs for extended periods prior to chase (5 or 10 min, lanes 9 and 10), the elongation rate was that expected for Pol II alone; that is, TFIIF stimulation of elongation was completely lost (Fig 1B). However, functional displacement of TFIIF by Gdown1 was surprisingly slow. Only half of the complexes lost TFIIF stimulation after a one minute incubation with Gdown1 (lane 7, Fig 1A; Fig 1B) and the near complete displacement of TFIIF was not apparent until at least five minutes (lane 9, Fig 1A; Fig 1B). To quantify the Gdown1 effect, we noted that essentially all of the TFIIF-stimulated complexes produced RNAs longer than ~120 nt under our conditions while in the absence of TFIIF stimulation, almost no RNAs were made longer than ~120 nt. Interestingly, there was very little effect of Gdown1 on the displacement of TFIIF when it was added with the chase NTPs (Fig 1A, lane 3).

**Fig 2:**
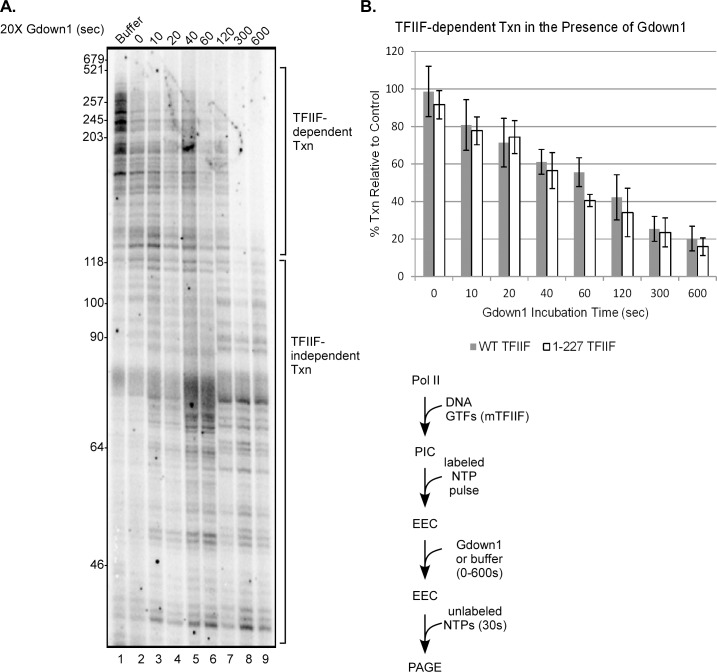
Truncation of the large subunit of TFIIF does not enhance functional Gdown1 loading to EECs. (A) PICs were assembled with 1–227 TFIIF and EECs were generated in solution on the AdML 31g(M) template with ATP, UTP and [α-^32^P]CTP as described in Materials and Methods. EECs were then incubated with 240 fmol of Gdown1 for 0 to 10 min (except lane 1) and chased for 30 sec with 200 μM NTPs. Lengths of markers are shown on the left edge of the gel, and lengths of TFIIF-dependent and TFIIF-independent transcripts are noted on the right. A schematic of the assay is shown to the lower right of the gel. (B) Average percents of TFIIF-dependent transcription (open bars) were quantified from results like those shown in panel (A) as described in Materials and Methods. The error bars indicate the mean ± S.D. based on four replicates. The grey bars duplicate the values from Fig 1.

Having established some basic parameters for the competition of Gdown1 and TFIIF in EECs, we wished to explore further the domains of TFIIF that affect that competition. TFIIF consists of two subunits, RAP30 and RAP74. The dimerization domains of these subunits, essential for TFIIF function, interact with Pol II on the Rpb2 subunit primarily on the lobe and protrusion domains [20–24]. Gdown1 has been reported to interact with that region of Rpb2 as well, but also with a number of other sites on Pol II [8,9]. Full TFIIF function requires only the 217 N-terminal residues of RAP74 [15]. Portions of the C-terminal segment of RAP74 from residues 218–517 are reported to extend into the central cleft of Pol II [23,24], but not all of this part of RAP74 has been located within Pol II transcription complexes [24,25]. To determine if this segment of RAP74 is involved in competing for Gdown1 interactions with Pol II, we repeated the set of experiments from Fig 1 using EECs halted at +30, but in this case the RAP74 subunit of TFIIF was severely truncated, retaining only the 227 N-terminal amino acids. As can be seen in Fig 2A and 2B, while TFIIF 1–227 is a slightly less efficient competitor of Gdown1, it is still fully capable of excluding Gdown1 from EECs. The time course of loading of Gdown1 into the 1–227 TFIIF EECs was very similar to that seen with full length TFIIF (Fig 2B).

**Fig 3:**
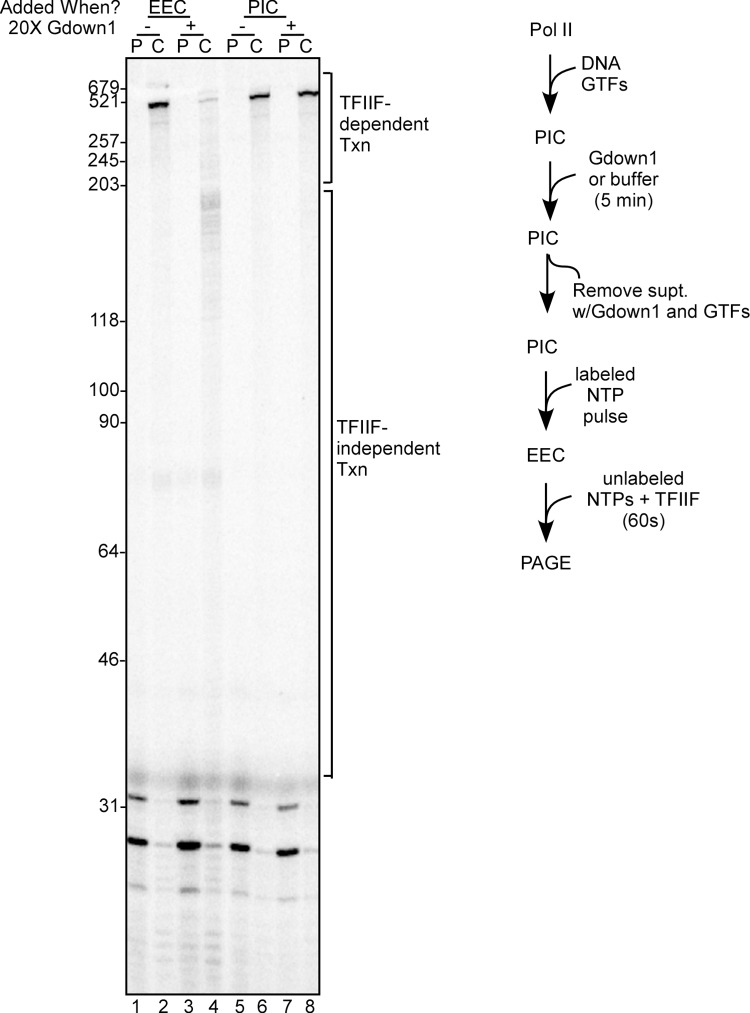
Gdown1 is unable to functionally associate with PICs. PICs were generated on bead bound AdML 31g(M) templates as described in Materials and Methods and then incubated for 5 min with 240 fmol of Gdown1 (lanes 7–8) or buffer (lanes 1–6). The supernatant was removed and replaced with buffer M5 and EECs were generated with pulse labeling as described in Materials and Methods. For lanes 3 and 4, EECs were then incubated with 240 fmol of Gdown1 for 5 min. At this point the reactions were stopped (P lanes) or supplemented with TFIIF and chased (C lanes) for 60 sec with 200 μM NTPs as described in Materials and Methods. Lengths of markers are shown on the left edge of the gel, and TFIIF-dependent and TFIIF-independent transcript lengths are noted on the right. A schematic of the assays in lanes 5–7 is shown to the right of the gel. The gel shown is representative of three replicates that were performed.

Our previous work had demonstrated that Gdown1 is unable to functionally enter a PIC [10], so we next sought to determine at what point in the transcription cycle the EEC is able to load Gdown1 in competition with TFIIF. Here we relied on the observations in Figs 1 and 2 that incubation of EECs with Gdown1 for 5 min should result in near-complete loss of TFIIF stimulated transcript elongation. To begin, we confirmed the inability of Gdown1 to enter PICs (Fig 3). PICs were incubated with Gdown1 for five minutes followed by removal of the supernatant, to exclude Gdown1 effects downstream of the PIC stage. Reactions were pulsed to +30 to generate EECs and chased with excess NTPs; in these experiments chases were for 60 sec, which allows Pol II to generate a 500 nt run-off transcript in the presence of TFIIF. As expected, there was no reduction in run-off RNA when Gdown1 was incubated with the PICs prior to chase (Fig 3, compare lanes 6 and 8). Gdown1 did prevent TFIIF stimulation of elongation when it was incubated for 5 min with a 30-mer EEC (compare lanes 2 and 4).

**Fig 4:**
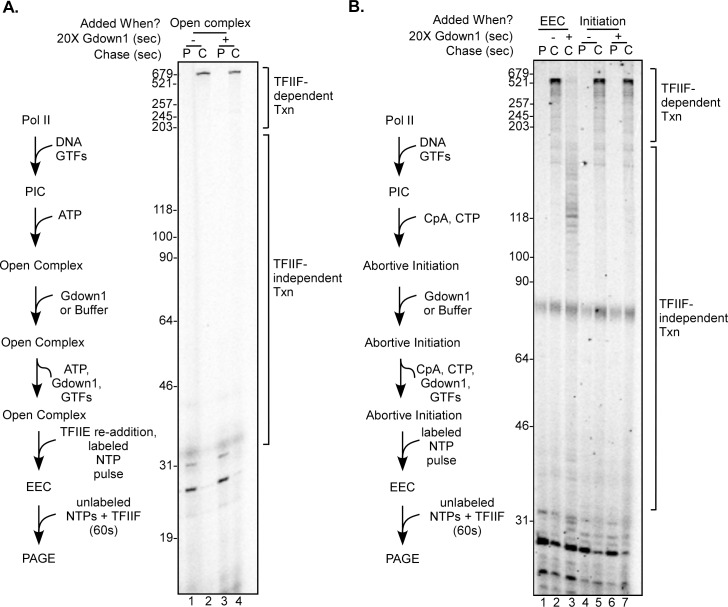
Gdown1 does not functionally associate with Pol II in open complex or with initiating Pol II. (A) PICs on bead bound AdML 31g(M) templates were converted to open complexes by incubation with ATP for 5 min, along with 240 fmole Gdown1 (lanes 3 and 4 only) as described in Materials and Methods. After the supernates were removed and replaced with buffer M5, reactions were supplemented with TFIIE and EECs were generated by pulse labeling with ATP, UTP and [α-^32^P]CTP as described in Materials and Methods. Reactions were either stopped at this point (P lanes) or supplemented with TFIIF and chased (C lanes) for 60 sec with 200 μM NTPs as described in Materials and Methods. Lengths of markers are shown on the left edge of the gel and TFIIF-dependent and TFIIF-independent transcript lengths are noted on the right. A schematic of the assay is shown to the left of the gel. The gel shown is representative of three replicates that were performed. (B) For lanes 4–7, complexes undergoing abortive initiation were generated on bead bound AdML 31g(M) templates with CpA and CTP as described in Materials and Methods. Reactions in lanes 6 and 7 contained 240 fmol of Gdown1. After 5 minutes the supernatant was removed and replaced with MEMDM40. EECs were then generated with ATP, UTP and [α-^32^P]CTP as described in Materials and Methods. Reactions were either stopped at this point (P lanes) or supplemented with TFIIF and chased (C lanes) for 60 sec with 200 μM NTPs. Control reactions shown in lanes 1–3 were performed as described in Fig 3 (EEC lanes). The lane 1 reaction was stopped after the pulse (P) step; for the two chase (C) reactions, Gdown1 was incubated for 5 min with the 30-mer complexes only for lane 3. Lengths of markers are shown on the left edge of the gel, and lengths of TFIIF-dependent and TFIIF-independent transcripts are noted on the right. A schematic of the assay in lanes 4–7 is shown to the left of the gel. The gel shown is representative of three replicates that were performed.

We next tested whether Gdown1 is able to functionally interact with transcription complexes following template melting or at initiation. The PIC is unique among transcription complexes in that the template DNA is entirely “outside” of the polymerase. We reasoned that the significant structural rearrangements that occur as the DNA is melted and the template strand is positioned near the active site might allow the onset of Gdown1 access to the transcription complex. To test the ability of Gdown1 to associate with an open complex we added ATP only to PICs and then repeated the Gdown1 challenge experiment as in Fig 3. The only modification to the protocol was that following the Gdown1 incubation, excess TFIIE was added to the reaction prior to supernatant removal. This is necessary because in the presence of ATP alone, transcriptional activity of the PIC rapidly decays. Our previous work demonstrated that additional TFIIE partially reverses ATP-mediated inactivation [26]. As seen in Fig 3 with the PIC, incubation of Gdown1 with an open complex did not result in loss of TFIIF stimulation of elongation (Fig 4A, compare lanes 2 and 4). We also asked if Gdown1 can enter a complex which is actually initiating transcription. We added the dinucleotide CpA plus CTP to open complexes, which we have shown results in extended synthesis of CAC [27]. (At AdML, the sequence around transcription start on the nontemplate strand is 5’CAC, where A is normally the first base of the transcript.) These abortively initiating complexes were incubated with Gdown1 using the same approach as in Figs 3 and 4A. Again, the addition of Gdown1 to initiating complexes failed to show any reduction in TFIIF-stimulated transcript elongation (Fig 4B, compare lanes 5 and 7). The positive control of Gdown1 acting on a 30-mer complex did show the expected loss of elongation stimulation by TFIIF (compare lanes 2 and 3).

**Fig 5:**
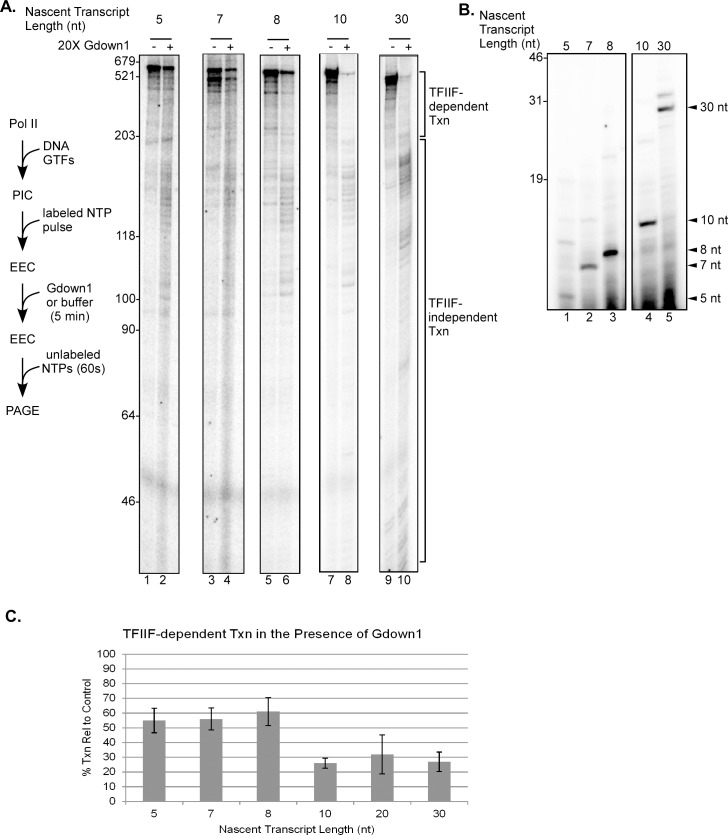
Efficiency of Gdown1 inhibition of TFIIF increases significantly with a 10 nt nascent RNA. (A) EECs were generated in solution on AdML 6g, 8g, 9g, 11g, and 31g(M) [19,30] templates with ATP, UTP and [α-^32^P]CTP as described in Materials and Methods. EECs were incubated with 240 fmol of Gdown1 or buffer for 5 min as indicated and then chased for 60 sec with 200 μM NTPs. Lengths of markers are shown on the left edge of the gel and TFIIF-dependent and TFIIF-independent transcript lengths are noted on the right. A schematic of the assay is shown to the left of the gel. Spaces in between lanes separate non-adjacent lanes within the same gel. In a separate experiment (gel not shown), reactions were performed as just described but using 21g templates [19] attached to beads to generate complexes with 20 nt RNAs. TFIIF-dependent and independent transcript lengths were determined as for the solution reactions. (B) 20% denaturing PAGE showing the nascent RNAs generated following the initial pulse. (Initial transcripts are not shown for the 21g bead-attached templates.) Arrows on the right indicate the expected transcript size relative to the G stop in the template. The space in between lanes 3 and 4 separates non-adjacent lanes within the same gel. (C) Average percents of TFIIF-dependent transcripts were quantified from results like those shown in panel (A) as described in Materials and Methods. The error bars indicate mean ± S.D. based on three to six replicates per individual template.

The results in Figs 3 and 4 demonstrate that Gdown1 is unable to enter a transcription complex at or before initiation. To determine how early in transcript elongation Gdown1 can enter the EEC, we generated complexes on a series of AdML templates with different lengths of G-less cassettes that allow the production of nascent transcripts ranging from 5 to 20 nt in the absence of GTP. We also included EECs with 30 nt RNAs as a control. The earliest EEC that will remain active during the necessary 5 min incubation with Gdown1 contains a 5 nt nascent transcript. As the RNA-DNA hybrid reaches 8–9 nt, the stability of the complex increases significantly [27,28]. Following incubation of the pulse labeled EECs with Gdown1 for 5 min, loss of TFIIF stimulation was assayed by chasing the complexes with unlabeled NTPs. As shown in Fig 5B, the GTP-less pulse labeling reactions produced primarily the expected 7, 8 and 10 nt nascent RNAs on the 8g, 9g and 11g templates. The most abundant RNA from the 6g template was a 5 nt transcript, but this template also supported a longer transcript, apparently 9 nt in length. We showed earlier that this longer RNA results from transcript slippage, not from read-through of the G stop [29,30]. In contrast to PICs and open or initiating complexes, all of the EECs did show significant reduction in TFIIF stimulated transcript elongation after a five minute exposure to Gdown1. However, we noticed a striking effect. The complexes that contained predominantly 5, 7, or 8 nt nascent RNAs retained nearly 60% of TFIIF-dependent transcription relative to the 30-mer controls (Fig 5A and 5C), while the complexes with 10 or 20 nt RNAs exhibited two to three-fold less TFIIF-stimulated elongation, a level similar to that seen with the 30-mer complexes. In the Fig 5 experiments the chases were for 60 sec. Substantial run-off RNA was made with the 5, 7 and 8 mer complexes in the presence of Gdown1 (lanes 2, 4 and 6) but this was not observed with the 10 and 30-mer complexes (lanes 8 and 10).

The substantial increase in the efficiency of functional Gdown1 interaction between the 8 and 10-mer complexes suggests a mechanistic explanation. A major structural transition occurs over this early stage of elongation, namely the reannealing of the extended upstream segment of the initial transcription bubble (bubble collapse) [16,30]. As we showed earlier, as Pol II advances from transcription start at the AdML promoter, the upstream edge of the transcription bubble remains anchored at its initial position and the bubble extends continuously until, at about +10, roughly the upstream half of the bubble abruptly reanneals [30]. This bubble collapse transition marks the end of the requirement for the XPB activity of TFIIH to assist elongation and the onset of promoter clearance [16,30]. The change in Gdown1 loading between +8 and +10 could result from this transition. However, the effect could simply be based on the presence of a longer transcript in the 10-mer complexes, relative to the 8-mer EECs.

**Fig 6:**
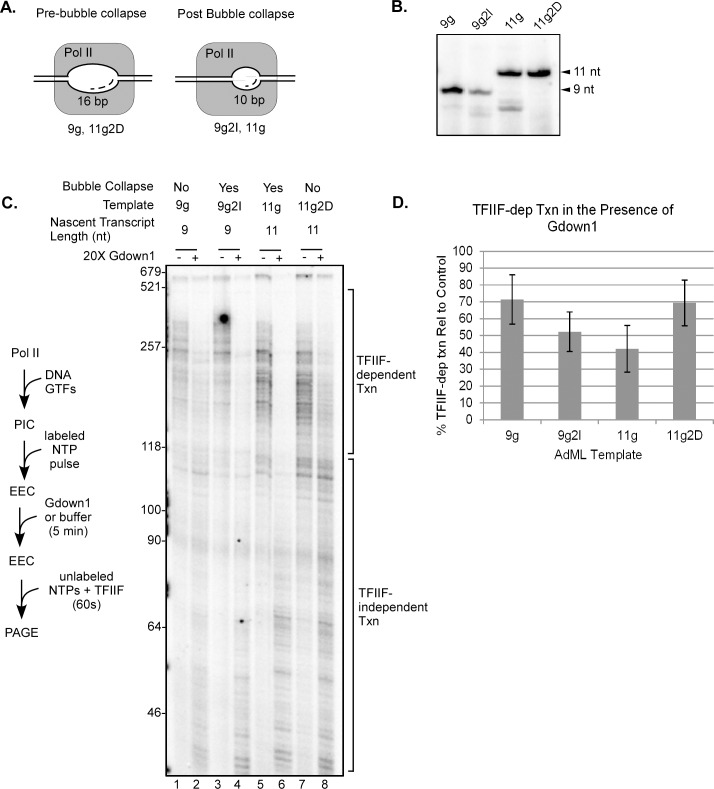
Functional binding of Gdown1 to EECs is enhanced following transcription bubble collapse. (A) Schematic showing the expected sizes of transcription bubbles for EECs paused at the initial G stops on the templates used in this figure (see [30]). (B) 20% denaturing PAGE showing the nascent RNAs generated following the initial pulse. Arrows on the right indicate the expected transcript size relative to the G stop in the template. (C) EECs were generated in solution on AdML 9g, 9g2I, 11g, and 11g2D templates with CpA, dATP, UTP, and [α-^32^P]CTP as described in Materials and Methods. EECs were then incubated with 240 fmol of Gdown1 or buffer for 5 min and chased for 30 sec with 200 μM NTPs. Lengths of markers are shown on the left edge of the gel and TFIIF-dependent and TFIIF-independent transcript lengths are noted on the right. A schematic of the assay is shown to the left of the gel. (D) Average percents of TFIIF-dependent transcription were quantified from results like those shown in panel (C) as described in Materials and Methods. The error bars indicate mean ± S.D. based on three to six replicates per individual template.

To discriminate between these two possibilities, we took advantage of the fact that bubble collapse, at least at AdML, occurs once the bubble reaches a maximum size (17–18 nt) regardless of transcript length [30]. Because the upstream edge of the bubble is positioned relative to the TATA box and not to transcription start, varying the distance from TATA to +1 generates templates on which bubble collapse will occur at different transcript lengths. We used two pairs of AdML-based templates that allow us to position EECs on either side of the collapse transition (Fig 6A). The 9g/9g2I template pair have the identical sequence from transcription start to the G-stop at +9 and therefore support synthesis of identical RNAs in the absence of GTP. However, on the 9g2I template two additional base pairs are present between the TATA box and transcription start. We showed that EECs halted at the G-stop on the 9g template have not undergone bubble collapse, while bubble collapse has occurred on EECs at the G-stop on 9g2I [30]. Similarly, the 11g and 11g2D templates support production of identical RNAs in G-less reactions. However, the 11g2D template has two fewer base pairs between TATA and transcription start, so complexes paused at the G-stop on 11g2D have not undergone bubble collapse (their bubbles are the same length as for complexes at the G-stop on 9g). Complexes halted at the G-stop on 11g have the identical RNA as complexes halted in G-less reactions on 11g2D, but they have passed the bubble collapse transition (analogous to the 9g2I complexes halted at the G-stop on that template [30]). Note that in these experiments we used the CpA dinucleotide primer, so that the 9g and 11g templates support the synthesis of 9 and 11 nt RNAs in the absence of GTP.

As shown in Fig 6B, the nascent RNAs produced in the initial G-less pulse reactions were primarily the expected lengths for all four templates. Complexes paused at the G stop were challenged with Gdown1 for five minutes and the effect on elongation was assayed by chasing with unlabeled excess NTPs. Remarkably, the two complexes paused prior to collapse (9g and 11g2D; Fig 6C and 6D) were only partially responsive to Gdown1, similar to the 5 through 8-mer complexes in Fig 5. Gdown1 had a greater effect on blocking elongation stimulation by TFIIF for the complexes paused after bubble collapse (9g2I and 11g; Fig 6C and 6D). In both cases the increased response to Gdown1 correlated with bubble size, but not with nascent transcript length. Finally, it should be noted that in our earlier study we showed that complexes paused at the G stop on the 6g template are clearly halted prior to bubble collapse, regardless of the presence of longer RNAs from transcript slippage [30]. This is consistent with idea that bubble collapse depends on reaching a maximum bubble size, regardless of RNA length. Importantly, it is also consistent with the fact that EECs on the 6g template respond to Gdown1 as expected for pre-collapse complexes (Fig 5A) even though RNAs up to 9 nt are produced by transcript slippage on that template. Overall, the results in Fig 6 demonstrate that the functional interaction of Gdown1 with the EECs is sensitive to the bubble collapse transition in early transcript elongation.

## Discussion

Previous work demonstrated that Gdown1 out-competes TFIIF for binding to free Pol II and Pol II in elongation-committed complexes [6,8–10]. However, despite this apparently universal ability of Gdown1 to bind tightly to Pol II, even in the presence of TFIIF, we were surprised to discover in our earlier work that Gdown1 cannot functionally interact with PICs [10]. Since Gdown1 is thought to play a role in promoter proximal pausing, this finding led us to a detailed study of the association of Gdown1 with Pol II complexes at and just downstream of transcription start. We now show that Gdown1 will not functionally enter the transcription complex at template unwinding or at initiation. Gdown1 will functionally displace TFIIF from the earliest EECs we can study, but this effect is surprisingly slow. Five minutes of incubation with Gdown1 were necessary to block TFIIF-stimulated elongation for all complexes that had achieved promoter clearance. An additional surprising point: Gdown1’s ability to associate with EECs is sensitive to a critical early transition. Gdown1 does not effectively displace TFIIF until EECs have undergone the bubble collapse transition.

The slow functional loading of Gdown1 to EECs might seem inconsistent with its expected role in promoter proximal pausing. In this context it is essential to note that while Pol II can travel as rapidly as 50–60 nt/sec within gene bodies [31–33], the half-life of promoter proximally paused Pol II in the nucleus has recently been estimated at 5–7 min [33,34]. Thus, during early elongation in vivo Gdown1 should have the opportunity to fully displace TFIIF. Pausing would be driven initially by other factors including DSIF, NELF and probably the first downstream (+1) nucleosome [1,35,36]. By preventing elongation stimulation by TFIIF and by synergizing with the other negative factors (such as GNAF [6]), Gdown1 would serve to lock promoter proximal Pol II in the paused state. Once resident in the paused complex, Gdown1 would also serve to block termination by TTF2 [6]. We have consistently observed very little effect of Gdown1 when it is added to EECs along with the chase NTPs. We have not attempted to quantify this effect, but a bias for Gdown1 to associate with paused versus freely-elongating transcription complexes would also be consistent with a primary role in retaining already-halted complexes in the paused state.

Once promoter proximal pausing is overcome, Pol II can enter into rapid, productive elongation. At roughly that point, Pol II associates with the Super Elongation Complex (SEC) collection of factors (reviewed in [37]). Interestingly, SEC subunits of the ELL class have been shown to have a TFIIF-like ability to stimulate Pol II elongation in vitro [38] (see also [39]), an effect which is enhanced by another class of SEC subunit, the EAF factors [38]. This suggests the intriguing possibility that a functional TFIIF substitute is embedded within the productive Pol elongation complex. Unlike the case with TFIIF, association of ELL/EAF with Pol II should not be blocked by Gdown1 since it has been shown that Gdown1 can reside in productive elongation complexes in vitro [6]. Retention of Gdown1 after the complex is released from promoter-proximal pausing might seem unexpected, but the continued presence of Gdown1 may be important in preventing termination by TTF2 during traversal of very long transcription units [6]. TTF2 can terminate Pol II across the entire genome, but only after Gdown1’s association with Pol II is weakened by phosphorylation at mitosis [40,41].

Gdown1 eventually displaces TFIIF from free Pol II and from all elongation complexes tested [6,8–10], but Gdown1 cannot stably associate with PICs [10]. We had speculated [10] that the structural changes accompanying template melting and initiation would be sufficient to enable Gdown1 to associate with the transcription complex, but that is clearly not the case (Fig 4). Identifying a structural basis for the inability of Gdown1 to load into PICs and newly-initiating complexes is problematic. An initial report on Gdown1-Pol II interactions identified a broad range of sites on the Rpb1, 2 and 5 subunits surrounding the central cleft in Pol II ([9]; see also [8]). A recent cryo-EM study of Pol II elongation complexes that apparently contained Gdown1 reported that it was not possible to locate Gdown1 at any particular position [42]. A recent comparison of the location of TFIIF in PICs, open complexes and EECs did not reveal major changes in TFIIF position among these complexes, at least for the portions of TFIIF that could be resolved [24]. Thus, it is currently unclear from a structural perspective why Gdown1 will only associate with the Pol II complex after elongation is underway.

The primary anchor for TFIIF on Pol II is centered on Rpb2 at/near the lobe and protrusion domains [20–24]. The locations of some portions of the C-terminal segment of RAP74 are not resolved in structural studies [24,25]. We can say that the residues from amino acids 228–517 of RAP74 do not play a major role in the TFIIF-Gdown1 competition (Fig 2). Our results are most consistent with the idea that competition for binding to the Rpb2 lobe and adjacent regions is the primary basis for Gdown1 to replace TFIIF on Pol II. It is worth recalling that TFIIF binds to Pol II with sufficient affinity that it was originally identified not as a GTF but as a Pol II binding factor [43]. This suggests that Gdown1 ultimately “wins” for binding to Pol II because Gdown1 can access multiple binding sites over the entire surface of Pol II surrounding the central cleft [9], potentially allowing Gdown1 to begin interactions outside the lobe and then eventually compete with TFIIF for lobe binding.

As shown in Figs 5 and 6, the efficiency with which Gdown1 is able to displace TFIIF increases dramatically following bubble collapse. Our results suggest that the collapse transition itself, and not transcript length per se, is the determining factor in this difference. Earlier work identified two potential connections between bubble collapse and the fate of other factors that could influence the TFIIF/Gdown1 competition. TFIIB transiently stabilizes TFIIF within the EEC [24,44]. However, TFIIB does not leave the elongation complex until +12/+13, several nt downstream of the collapse transition [24,44,45]. The activity of the XPB subunit of TFIIH is no longer required for effective elongation once the upstream segment of the initial transcription bubble reanneals [30,46] and thus it is possible that XPB disengages from the leading edge of the transcription complex at that point. Even if loss of TFIIH interactions do occur at bubble collapse, the most recent structural studies do not identify interactions between TFIIH and the rest of the EEC near regions thought to be important for TFIIF and Gdown1 binding [24]. Therefore, neither loss of TFIIB-TFIIF interactions nor potential loss of contacts of TFIIH with the elongation complex seem likely as explanations for the effect of bubble collapse on the interaction of Gdown1 with the early elongation complex.

Regardless of the structural basis, our results indicate that for the AdML promoter, all transitions in the nascent elongation complex that might affect Gdown1 loading should occur well upstream of the typical promoter proximal pause location at roughly +50 [1]. However, consensus TATA box promoters such as AdML represent less than 10% of mammalian Pol II promoters [47]. It is not known where the promoter clearance/bubble collapse transition occurs for non-TATA promoters. Transcript initiation at such promoters is typically supported over a wide range of possible locations [47,48]. In yeast, Pol II transcription bubbles at promoters can extend up to 80 bases downstream from the initial point of DNA unwinding [49,50]. It is conceivable that over the entire spectrum of TATA-less mammalian promoters, the bubble collapse transition may occur much farther downstream than is typical for TATA promoters, in some cases relatively close to the major pause location and the +1 nucleosome. Thus, the effectiveness of Gdown1 loading at the promoter-proximal pause could be a sensitive function of promoter architecture and the relative position of the +1 nucleosome.

In conclusion, our results support a model in which Gdown1 functions in vivo by associating with EECs following an initial promoter-proximal pause mediated by other factors. Loading of Gdown1 would displace the elongation-stimulating TFIIF factor, thereby helping to lock the complex in a paused state resistant to termination by TTF2. The maintenance of extended pausing in vivo is important for the regulation of gene expression, not only by providing a mechanism for the rapid induction of transcription in response to regulatory signals but also by maintaining promoter regions in an accessible state by blocking competitive nucleosome assembly [51]. Given that association of Gdown1 with the nascent transcription complex is sensitive to promoter clearance, differences in promoter architecture may be important in the relative ability of Gdown1 to load as EECs enter into the paused state downstream of transcription start.

## Materials and Methods

### Reagents

Reagents were sourced from the following: DNA primers from Integrated DNA Technologies, NTPs from New England Biolabs, CpA from TriLink, RNasin Plus from Promega, Streptavidin-coated M280 Dyna Beads from Invitrogen, and 800 Ci/mmol [α-^32^P]CTP from PerkinElmer Life Science and MP Biomedicals.

### DNA templates

All templates contained the Adenovirus major late TATA box promoter, with G-free segments on the nontemplate strand extending from 5 to 30 bp downstream of transcription start [19,30]. Overall length of the DNAs was 600 bp with 100 bp upstream of the transcription start site. The templates were generated by PCR amplification from plasmid DNA and purified using the QIAQuick PCR Purification Kit (Qiagen). The upstream primer was biotinylated to allow attachment to streptavidin-coated beads. The template names all include the location of the G stop; for example, in the absence of GTP, 9g-class templates support the synthesis of 8-mer RNAs (or 9-mer RNAs, if initiation is primed with CpA). The template pairs chosen for the Fig 6 study allow the comparison of complexes with identical transcripts that have not yet reached, or have passed through, the bubble collapse transition. The 9g2I template has two additional bp added between TATA and +1, relative to 9g; 2 bp were removed from the 11g2D template between TATA and +1, relative to 11g. Complexes halted in the absence of GTP on 9g and 9g2I have the identical nascent RNAs, but because of the insertion in 9g2I, complexes on that template have undergone bubble collapse, while bubble collapse has not occurred for G-stop complexes on 9g. Similarly, both 11g templates support identical transcripts in the absence of GTP, but G-stop complexes on 11g have undergone bubble collapse while collapse has not occurred for G-stop complexes on 11g2D (see [30]).

### Proteins and factors

Recombinant human TATA box binding protein (TBP), TFIIB, and TFIIE were purified as described [30,52]. Recombinant human TFIIF subunits, including both full length RAP74 and the truncated 1–227 RAP74, were expressed, purified, and assembled as described [17]. TFIIH was purified from HeLa nuclear extract by chromatography on P11 phosphocellulose and DE52 [17]. RNA polymerase II was purified from chromatin pellets obtained during preparation of HeLa nuclear extract with chromatography on DE52, heparin Sepharose, and Mono Q. This method was modified from that of Maldonado et al. [53] as described [52]. Recombinant His-tagged human Gdown1 was purified as described [6] on nickel-nitrilotriacetic acid resin followed by chromatography on Mono Q. It was stored and diluted in 25 mM HEPES, pH 7.6, 20% glycerol, 300 mM KCl, 0.1 mM EDTA, 1 mM DTT, and 0.1% PMSF with 10 μg/ml BSA.

### Assembly of preinitiation complexes

Preinitiation complexes were assembled for 20 minutes at 30°C. Each 10 μl reaction included the following: 50 ng of template DNA (either bead attached or in solution), 0.4 μl of Pol II (~6 ng, ~12 fmol), 95 fmol of TBP, 72 fmol of TFIIB, 72 fmol of TFIIF, 3.3 fmol of TFIIE, and 0.5 μl of TFIIH (DE52 fractions, ~75% saturation of activity). The final buffer conditions for PIC assembly were as follows: 20 mM Tris-HCl (pH 7.9), 60 mM KCl, 8 mM MgCl_2_, 2 mM DTT, and 0.12 mg/ml BSA. For some experiments, bead-bound PICs were incubated with buffer or 240 fmol of Gdown1 for 5 minutes at room temperature. (240 fmol of Gdown1 represents a 20-fold molar excess over Pol II.) Following incubation, the supernatant was replaced with M5 (20 mM Tris-HCl, pH 7.9, 0.25 mM EDTA, 65 mM KCl, 10 mM β-glycerophosphate, 10 mM MgCl_2_, 1 mM DTT, and 10 μg/ml BSA) prior to pulse-labeling and chase.

### Generation of pulse-labeled early elongation complexes

Our various AdML-based templates have non-template strands in which the first G residue downstream of transcription start is located from +6 to +31, as noted in the figure legends. To generate EECs, PICs were incubated in 10 μl reactions with the following: for Figs 1, 2, 3, 4 and 5: 500 μM ATP, 50 μM UTP, 0.71 μM [α-^32^P]CTP and 0.5 units/μl RNasin plus; for Fig 6, 1 mM CpA, 20 μM dATP, 10 μM UTP, 0.71 μM [α-^32^P]CTP and 0.5 units/μl RNasin plus. In all cases reactions were incubated for 2 minutes at 30°C followed by addition of 50 μM CTP for 30 seconds at 30°C to guarantee advancement of the nascent RNAs to the first downstream G stop. To test for the ability of Gdown1 to functionally associate with these complexes, 240 fmole of Gdown1, or buffer as a control, were incubated with each reaction for 0–10 minutes at room temperature prior to chase with nonlabeled NTPs. Some reactions were stopped prior to chase to assess the lengths of the pulse labeled RNAs; these transcripts were purified by extraction with phenol and CHCl_3_ and resolved by denaturing PAGE.

### Generation of open complex

To assess the ability of Gdown1 to functionally associate with open complexes, PICs assembled on bead-attached AdML 31g(M) templates were incubated with 100 μM ATP and 240 fmol of Gdown1 or buffer for 5 minutes at room temperature. The supernates with ATP and Gdown1 were removed and replaced with buffer M5. To prevent loss of activity during open complex formation, 13 fmol of TFIIE were added to each reaction before pulse labeling [26]. Early elongation complexes (EECs) were then generated by incubation with 500 μM ATP, 50 μM UTP, 0.71 μM [α-^32^P]CTP, and 0.5 units/μl RNasin plus for 2 minutes at 30°C followed by addition of 50 μM CTP for 30 seconds at 30°C to guarantee advancement of the nascent RNAs to the first downstream G stop.

### Abortive initiation

To assess the ability of Gdown1 to functionally associate with complexes at the point of initiation, we incubated PICs assembled on bead-attached AdML 31g(M) templates with substrates allowing the formation of only the first bond, The sequence of the nontemplate strand of the AdML templates near the transcription start site is 5’CACT (underlined A residue is +1). 1 mM CpA, 25 μM dATP, and 1 μM CTP were added to PICs for 5 minutes with either buffer or 240 fmol of Gdown1 to generate complexes undergoing continuous abortive initiation [27]. To end abortive initiation and remove Gdown1, the supernatant was removed and replaced with MEMDM40 (30 mM Tris, pH 7.9, 40 mM KCl, 10 mM β-glycerophosphate, 0.5 mM EDTA, 10% glycerol, 8 mM MgCl_2_, 1 mM DTT) with 10 μg/ml BSA. Early elongation complexes were then generated by incubation with 500 μM ATP, 50 μM UTP, 0.71 μM [α-^32^P]CTP, and 0.5 units/μl RNasin plus for 2 minutes at 30°C followed by incubation with 50 μM CTP for 30 seconds at 30°C to guarantee advancement of the nascent RNAs to the first downstream G stop.

### Transcript elongation

EECs prepared as described above were chased with 200 μM NTPs (ATP, CTP, GTP, and UTP) at 30°C for 30–60 seconds, as indicated in the figure legends. Reactions in which Gdown1 was incubated with PICs, open complexes, or abortively initiating complexes also had 95 fmol of TFIIF added with the chase. When Gdown1 was added at the zero time point, Gdown1 was combined with the chase NTPs just prior to addition to the reaction. Reactions were stopped by the addition of buffer containing 10 mM Tris-HCl (pH 7.9), 100 mM NaCl, 10 mM EDTA, 1% sarkosyl, and 0.2 mg/ml tRNA. Transcripts were purified by extraction with phenol and CHCl_3_ and resolved by denaturing PAGE.

### Quantitation of TFIIF-dependent and TFIIF-independent transcription

All gels were imaged with a Typhoon Trio and gel quantitation was performed with ImageQuant TL software. The fractions of TFIIF-dependent transcription (larger than 120 nt in 30 second chases, or larger than 200 nt in 60 second chases) were calculated in Microsoft Excel from the total transcription, and then normalized to the fraction of TFIIF-dependent transcription in the buffer control reactions. The percent of TFIIF-dependent transcription in the presence of Gdown1 relative to the buffer control reactions was then plotted. Each plotted value represents at least three independent replicates.
